# Metatypical basal cell carcinoma: a clinical review

**DOI:** 10.1186/1756-9966-27-65

**Published:** 2008-11-07

**Authors:** Mauro Tarallo, Emanuele Cigna, Riccardo Frati, Sergio Delfino, Daniele Innocenzi, Umberto Fama, Annamaria Corbianco, Nicolò Scuderi

**Affiliations:** 1 Department of Dermatology and Plastic Surgery, University "La Sapienza", Rome, Italy

## Abstract

**Background:**

Metatypical cell carcinoma can be considered as a new entity of skin cancer, being an intermediate typology between basal cell carcinomas and squamous cell carcinomas. The behaviour of the metatypical cell carcinoma lies between these two varieties of skin cancer. It is difficult to perform a differential diagnosis based on morphological and clinical features – therefore it is only possible by accurate histology.

**Methods:**

The authors have retrospectively analysed clinical records of 240 patients who were affected by metatypical skin cancer and who were treated by surgery, radiotherapy and chemotherapy.

**Results:**

MTC affected more males than females (62.5% vs 37.5%) than males. The most affected site was the cervicofacial area, 71.7%; then the trunk, 10%; the limbs, 9.6%; the scalp 3.7%; and other regions 5%. A recurrence occurred in 24 cases (10%), mainly in head and neck area.

**Conclusion:**

In this manuscript, the authors have emphasised the importance of conducting a differential diagnosis, and the importance of the specific treatment for metatypical skin cancer, even though more clinical studies and long-term follow-ups are required before establishing specific guidelines.

## Background

Nonmelanoma skin cancers are the most common type of cancer, with over 1.3 million new cases diagnosed and treated annually, in the Western Coutries alone, which indicates an increasing prevalence [1-4]. Non-melanoma skin cancer is composed of different tumours, 95% of which consist of basal or squamous cell carcinoma [1-4].

Although advances in molecular genetics have localised mutations for numerous nonmelanoma skin cancers, the cause remains multifactorial [2-5]. Moreover, environmental (U.V. radiation) and lifestyle factors, as well as the aging population, certainly play a key part in the onset of a tumour [2-4]. Although skin cancers are more common in Caucasians, they are also prevalent in people with a dark complexion (e.g. African-Americans) [6-9].

Basal cell carcinoma tends to develop on the head and neck, and other sun-exposed areas, whereas squamous cell carcinoma has a strong link with advanced age (i.e. >40 years), and ultraviolet exposure. They are commonly present on non-facial sites and have a propensity to develop in sun-exposed areas of the body. Both basal cell and squamous cell carcinomas may, however, also develop on non-sun-exposed areas [1-3].

Basosquamous carcinoma, also known as metatypical carcinoma (MTC), is a non-melanoma skin cancer that shares the features of both the squamous and basal cell carcinomas. This tumour should be considered as another skin cancer, with its own particular characteristics, such as behaviour and histological features.

As it is an intermediate typology between basal cell carcinoma (BCC) and squamous cell carcinoma (SCC), the metatypical carcinoma simulates the BCC clinically and morphologically, but compared with BCC it is more aggressive and prone to metastasise [10-15]. Therefore, the separation of MTC from the group of basaliomas is of primary importance, as this tumour is capable of metastasizing, whereas MTC diagnosis is difficult because it is similar to basalioma clinically and hystologically [10-12].

The definition of the basosquamous cell carcinoma and the presence of intermediate areas of differentiation of this tumour have been emphasised by some authors, and it has been suggested that metastatic-basal-cell carcinoma and MTC may be the same tumour [13-17].

However, there are some studies about the mitotic rate – in cases of MTC and BCC cases of basalioma – that have shown this finding to be adequate for differential diagnosis of these tumours. The values of the mitotic regimen in MTC differ from the similar types of values in the basalioma: the mitotic activity; the specific content of dividing cells at the stage of metaphase; and the rate of pathologic mitoses increased considerably [13-17].

MTC, however, has been poorly defined clinically and pathologically, and a result has no general acceptance in medical literature [18-24].

Concerning the surgical treatment, it has been discovered that a significant proportion of excised BCC demonstrates histological positive-surgical margins. This high incidence of positive-surgical margins for excised BCC may be caused by the irregular infiltration of these tumours. As a result, the surgeon cannot clinically detect the subclinical spread. So, the inadequate excision of a BCC and of the MTC that is clinically similar is possible. Furthermore, the more appropriate margin is still controversial for the BBC, as it is for the MTC.

**Figure 1:**
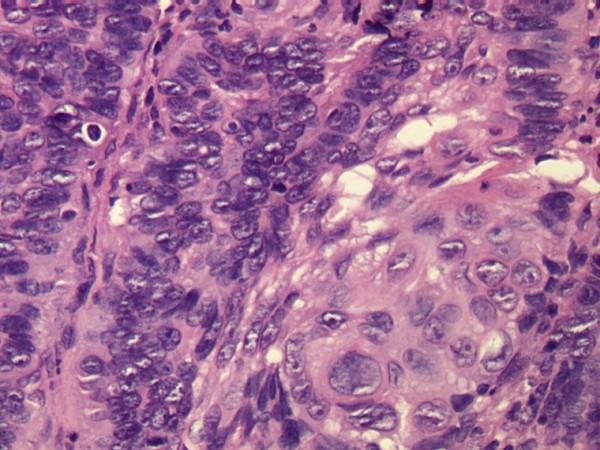
**Histological appearance of a metatypical cell carcinoma**. A: Mixed type hematoxylin eosin stain 20 ×. B: Intermediate type hematoxylin eosin stain 20 ×.

**Figure 2:**
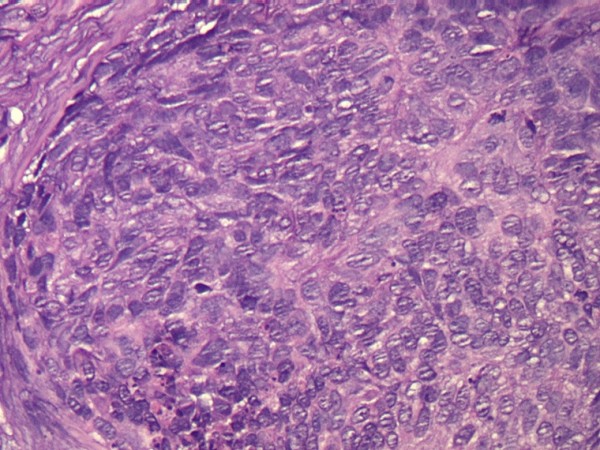
**Histological appearance of a metatypical cell carcinoma**. Intermediate type hematoxylin eosin stain 20 ×.

We retrospectively reviewed the cases of MTC-operated patients during a seven-year period (1996 to 2003) at the Department of Plastic Surgery of University of Rome "La Sapienza", to find the rates of recurrence and metastasis of these lesions within this group, and to see whether or not the histological presentations (mixed type and intermediate type [Figures 1 and 2, respectively]) were more apparent in a particular subgroup of age or sex.

## Materials and methods

Another 240 patients with cases of MTC affecting different regions (the trunk, upper and lower limbs, scalp, neck, and facial), were retrospectively analysed from 1996 to 2003 at the Department of Plastic Surgery of the University of Rome "La Sapienza". The study included 90 females and 150 males, between the ages of 27 and 95. The average age was 70.5 years.

All tumours were measured and excision margins were marked. The borders were marked on the basis of visual or palpable alterations on surface contours, consistent with a non-melanotic skin carcinoma. The minimum surgical margin was that of the short axis of the ellipse. Tumour size, location, and the availability of loose donor skin at the surgical margins were taken into account to help determine the appropriate surgical margin for each tumour, and to provide an ideal surgical closure while clearing the tumour in a single excision.

Ellipses were designed as an eccentric parallelogram before infiltration with local anaesthesia and without stretching the skin. A precise margin of apparently healthy skin was taken around the outer border of the ink marking the tumour: Peripheral clearance margins of 3 mm around tumours in facial areas and 5 mm margins around lesions in other sites. Although, in cases with a clinical history of rapid growth, in which a standard 5 mm surgical margin have been adopted for head and neck lesions and 10 mm for other regions.

Full-depth dermal incisions, perpendicular to the skin surface, were made along the outer inked edge of each marked ellipse. These ellipses were removed beneath the dermis.

A histological determination was made to determine whether there was a tumour present at the surgical margins. If a tumour was present, additional skin was removed and histologically verified until clear margins were obtained.

Diagnosis was obtained by histology. Management foresaw surgical excision, radiotherapy and chemotherapy. A follow-up was conducted up to five years after surgery.

## Results

In this study, MTC affected more males than females (62.5% vs 37.5%). The most affected site was the cervicofacial area, 71.7% (172 cases); then the trunk, 10% (24 cases); the limbs, 9.6% (23 cases); the scalp 3.7% (9 cases); and other regions 5% (12 cases) (Table 1). In all cases, diagnosis of MTC was confirmed.

**Table 1:** Patient's population group and areas affected by MTC.

**Areas**	**Males**	**Females**	**tot**
**Cervico-facial**	107 (44,6%)	65 (27,1%)	172 (71,7%)

**Trunk**	16 (6,6%)	8 (3,3%)	24 (10%)

**Limbs**	14 (5,8%)	9 (3,7%)	23 (9.6%)

**Scalp**	5 (2%)	4 (1,6%)	9 (3.7%)

**Other regions**	8 (3,3%)	4 (1,6%)	12 (5%)

**tot**	150 (62,5%)	90 (37,5%)	240 (100%)

The average diameter of the lesions was 1.3 cm, the largest tumour measured 5 × 3 cm, and the smallest 0.6 × 0.4 cm.

The margins differed based on the anatomic location and on the growth pattern based on clinical history. The mean surgical margin was 3 to 5 mm on the facial area and 5 to 10 mm on the other areas. The wider excision in cases of incomplete eradication of a MTC was 5 mm around the entire scar of the previous surgery (Table 2).

**Table 2:** Surgical margins adopted for MTC excision.

	**Standard**	**Rapid-growth history**
**Primary excision H&N**	3 mm	5 mm

**Primary excision body**	5 mm	10 mm

**Wider excision**	5 mm	5 mm

Histological examination showed different subtypes such as mixed (32%), intermediate (68%). Ulceration was occasionally present (10%) and an infiltrative aspect has been observed in 2.5% of patients. 62% of patients with mixed metatypical carcinoma were males, with an average age of 68.6 years, and 38% females with an average age of 71.8 years. Intermediate metatypical carcinoma was found in 68% of cases, striking mainly males (66%) with an average age of 69.6 years, and 34% females with an average age of 72.7 years (Table 3). A recurrence occurred in 24 cases (10%), mainly in head and neck -these were re-operated using a wider excision (Table 3).

**Table 3:** histological types of MTC detected

**Type**	**n°**	**%**
**intermediate**	108	45%

**mixed**	55	23%

**ulcerated**	24	10%

**infiltrative**	6	2.5%

**non specified**	47	19,5%

## Discussion

The incidence of non-melanoma skin cancer varies based on geographic location, with the highest rate of 1 to 2% per year in regions of high ultraviolet exposure, such as Australia [3]. Among the more common BCC and SCC, the MTC has different rates of local recurrence, disease spread and mortality. Differential diagnosis between MTC and the group of basaliomas is difficult for the similar clinical findings, but it is imperative for the risk of metastases associated with the MTC if compared to BCC [6-8,10-15]. Then MTC should be considered as another entity of non-melanoma skin cancer, as intermediate typology between BCC and SCC.

In our study group we found more lesions on the head and neck area (71.6%) compared to other areas like the trunk (10%) or the limbs (9.6%), scalp (3.8%), and other zones (5%). Among these cases the intermediate metatypical carcinoma was found in 68% of cases, striking mainly younger males (66%; average age of 69.6 years) than females (34%; average age of 72.7 years), whereas the mixed metatypical carcinoma was found in 32% of cases, 62% were males (average age of 68.6 years), and 38% females (average age of 71.8 years). We can confirm in our study that MTC was more prevalent in old ages and facial/head-neck areas as SCC, but there was no significant predilection of histological type for any sex in our study, as it was not a representative population.

In an another study, mixed metatypical carcinoma was found in 24% of cases, with an average age of 71 years, and prevalence on head (81%); while intermediate metatypical carcinoma, was found in 76% of cases, striking mainly the head (70%), then the trunk (19%) and limbs (9%). These findings are similar to our results.

Concerning the surgical excision margins to be adopted for MTC excision, we reviewed the literature of the BCC and SCC standard surgical margin and recurrence rate risk.

Histological positive surgical margins of excised BCC are considerably high (>16% for head and neck region) [25-30]. Perhaps, some reports show very high recurrence rates, reaching up to 52% of inadequate excision of all BCC excised [31,32]. Lesions in the temporal and forehead areas are particularly prone to recur, or metachronous basal cell carcinoma may occur in these areas [33]. So, clinically, for BCC and SCC larger peripheral margins are marked on head and neck areas to avoid recurrence and then increase the risk of disease spread.

Studies report wide ranges of surgical margins, ranging from 2 to 10 mm or more, for BCC due to the clinical difficulties in judging the margins of basal cell carcinomas [34,35]. Different studies with a 3 to 5 mm margin for primary BCC excisions report incomplete excisions of about 4% for either basal cell or squamous cell carcinoma, only in cases with clear clinical tumour margins as for the nodular basal cell carcinoma [36].

Other studies have found a considerably higher percentage of lesions that need a wider margin, while Goldberg [37,38] recommends 2 to 5 mm surgical margins, and 10 mm for infiltrative lesions.

For SCC, the anatomic locations influenced tumour aggressiveness, and the reconstructive surgery possibilities. The mucosal variant requires special attention because of its high propensity to recur and metastasise [25]. Squamous cell carcinoma of the ear also requires a special mention, as it represents one of the most common origins for metastasis, and is the anatomic site with the highest rate of recurrence (18.7%) [26]. Tumour thickness has also been shown to correlate with metastatic rates [25,26].

Although no satisfactory reports have been published on squamous cell carcinoma, in terms of an optimal margin to predict recurrence rates, subsets of squamous cell carcinoma may still recur, despite having a complete excision [39]. For SCC the degree of cellular differentiation represented by keratinisation has been correlated with tumour aggressiveness, as poorly differentiated squamous cell carcinoma has a reported recurrence rate of 28.6%, and metastatic rate of 32.8%, where as well-differentiated squamous cell carcinoma is 13% and 9.2%, respectively [40].

The suggested standard surgical margin for primary nonmelanoma skin cancers is 4 mm [36,41,42]. This surgical margin of 4 mm would have achieved an optimal excision in 96% of basal cell and 97% of squamous cell carcinoma [36,41,42]. However, studies have shown that only 7% of small, well-circumscribed primary BCCs, infiltrate beyond 1 mm of their clinical margins [43], thus being in agreement with Asadi and colleagues [36].

Based on these studies, and on clinical experience with SCC and BCC, we adopted a surgical margin of 3 to 5 mm for the head and neck lesions and of 5 to 10 mm in the other areas, using the wider margin in cases of rapid growth clinical history.

Having clear histological margins does not always guarantee that a tumour will not reappear because the presence of discontinuous subclinical tumour extension could give rise to tumour recurrence [44]. In these cases, neither conventional surgery [45] nor Mohs' micrographic surgery [46,47] can be expected to resect such discontinuous tumours, unless fortuitously wide margins of conventional surgery clearance include such tumour discontinuities. A prospective randomised study of local recurrence after both techniques did not show that one method was statistically superior to the other during a 30-month follow-up period [34].

The recurrence rate of our study group for a 3–5 mm and a 5–10 mm surgical excision, occurred in 24 cases (10%), these were re-operated with a wider excision until obtaining free of disease surgical margins. This could lead us to confirm the tendency of MTC to have the aggressiveness of SCC.

Although mortality rates are low for both, they are significantly higher for squamous cell carcinoma than for basal cell carcinoma. Mortality from squamous cell carcinoma is frequently secondary to metastases of tumour originating from the ear [48].

In addition to the morbidity and mortality associated with squamous cell carcinoma of the ear, squamous cell carcinoma of the lip carries the highest rate of metastasis (13.7%) [49].

Basal cell carcinoma is rarely metastatic, with a reported incidence of 0.0028% to 0.55% [13,14]. Although variable, the risk of metastasis for squamous cell carcinoma is greater, estimated at approximately 5%, with a range of 0.5% to 6%, and some reports reaching 16% [50-52].

The metatypical carcinoma is aggressive and metastatic, with rates of metastasis reported up to 7.4%, in between the SCC and the BCC metastatic rate [53-55].

In our five-year follow up, mortality did not occur, but four patients (4/240 1,6%) needed positive regional lymph node dissection.

## Conclusion

MTC requires a different management compared to that of the BCC, particularly if it has been incompletely excised. However, these differential diagnoses can occasionally pose difficult morphological problems. The stated distinctions are clinically important because the risk of progressive disease is significantly higher with squamous carcinoma of the skin and MTC than of the BCC.

The excision should be wider than the ones adopted with BCC's excision, particularly as the lesion has a history of fast growth. However, in medical literature, no guidelines regarding MTC excision margins have been established yet. On the basis of our experience, we believe that a wider excision should be adopted when dealing with a MTC that has been histologically proved, and a special follow up should be conducted. As a clinical differential diagnosis cannot be surely achieved, it is more appropriate to avoid a wide excision and skin sacrifice, especially with facial areas. Due to MTC having a higher growth rate than BCC, an adequate excision for early onset lesion is necessary. The MCC diagnosis and treatment in the early stages can lead to a satisfactory recovery.

However, further clinical studies with long-term follow-up will still be required to clear any doubts the management of the MCC may have.

## Competing interests

The authors' declare there no financial competing interests (political, personal, religious, ideological, academic, intellectual, commercial or any other) with the study.

## Authors' contributions

MT main author, project of the study, EC interpretation of data, RF data analysis, SD data analysis, DI histology analysis, UF patient's data collection, AC patient's data collection, NS study coordinator.

## References

[B1] Miller SJ (2004). The impact of non-melanoma skin cancer. J Nat Comp Cancer Net.

[B2] Strom SS, Yamamura Y (1997). Epidemiology of nonmelanoma skin cancer. Clin Plast Surg.

[B3] Diepgen TL, Mahler V (2002). The epidemiology of skin cancer. Br J Dermatol.

[B4] Zak-Prelich M, Narbutt J, Sysa-Jedrzejowska A (2004). Environmental risk factors predisposing to the development of skin cancer. Dermatol Surg.

[B5] Housman TS, Williford PM, Feldman S (2003). Nonmelanoma skin cancer: an episode of care management approach. Dermatol Surg.

[B6] Halder RM, Bridgeman-Shah S (1995). Skin cancer in African-Americans. Cancer.

[B7] Halder RM, Bang KM (1988). Skin cancer in blacks in the United States. Derm Clin.

[B8] Mora RG, Perniaciaro C, Lee B (1984). Cancer of the skin in blacks: a review of nineteen black patients with Bowen's disease. J Am Acad Dermatol.

[B9] Kwa RE, Campana K, Moy RL (1992). Biology of cutaneous squamous cell carcinoma. J Am Acad Dermatol.

[B10] Innocenzi D, Francesconi L, Ruggiero A, Potenza MC, Proietti I, Nicolucci F, Skroza N, Soda G (2006). Il carcinoma metatipico della cute. Derm Clin.

[B11] Kazantseva IA, Bogatyreva II, Vavilov AM, Parshikova SM (1983). Differential diagnosis of basalioma and metatypical skin cancer]. Arkh Patol.

[B12] Konrad EA, Wolburg H (1983). Metatypical carcinoma of the lower eyelid. Ophthalmologica.

[B13] Kazantseva IA, Khlebnikova AN, Babaev VR (1996). Immunohistochemical study of primary and recurrent basal cell and metatypical carcinomas of the skin. Am J Dermatopathol.

[B14] Kazantseva IA, Khlebnikova AN, Babaev VR, Fager G (1994). [Proliferative activity of basal cell and metatypical cell carcinoma of the skin]. Arkh Patol.

[B15] Reis-Filho JS, Torio B, Albergaria A, Schmitt FC (2002). Maspin expression in normal skin and usual cutaneous carcinomas. Virchows Arch.

[B16] Swanson PE, Fitzpatrick MM, Ritter JH, Glusac EJ, Wick MR (1998). Immunohistologic differential diagnosis of basal cell carcinoma, squamous cell carcinoma, and trichoepithelioma in small cutaneous biopsy specimens. J Cutan Pathol.

[B17] Barrett TL, Smith KJ, Hodge JJ, Butler R, Hall FW, Skelton HG (1997). Immunohistochemical nuclear staining for p53, PCNA, and Ki-67 in different histologic variants of basal cell carcinoma. J Am Acad Dermatol.

[B18] Tosca A, Varelzidis A, Bassioukas K, Hatzis J, Nicolis G, Stratigos J (1985). Some further features for differential diagnosis between squamous cell carcinomas and basal cell epitheliomas. Dermatologica.

[B19] de Faria JL, Navarrete MA (1991). The histopathology of the skin basal cell carcinoma with areas of intermediate differentiation: A metatypical carcinoma?. Pathol Res Pract.

[B20] Sari A, Basterzi Y, Yavuzer R, Latifoglu O (2002). Linear Nevus Sebaceus Complicated with Metatypical Carcinoma. Plast Reconstr Surg.

[B21] Hirschsteiner O, Maiwald G, Balda BR (2000). Guess what! Diagnosis: Extended ulcerating metatypical basal cell carcinoma (BCC) with soft tissue and bone destruction. Eur J Dermatol.

[B22] Gall C, Buttner A, Bise K, Steiger HJ (1997). Primary intracranial metatypical basal cell carcinoma: case report. Neurosurgery.

[B23] Labbe D, Lample GD, Rigot-Jolivet M, Compere JF, Joly F, Mandard JC (1994). [Metatypical carcinoma. Apropos of 4 cases]. Ann Chir Plast Esthet.

[B24] Bianchini R, Wolter M (1987). Fatal outcome in a metatypical, giant, "horrifying" basal cell carcinoma. J Dermatol Surg Oncol.

[B25] Bucur A, Stefanescu L (2004). Management of patients with squamous cell carcinoma of the lower lip and neck. J Craniomaxillofac Surg.

[B26] Chu A, Osguthorpe JD (2003). Nonmelanoma cutaneous malignancy with regional metastasis. Otolaryngol Head Neck Surg.

[B27] Niederhagen B, von Lindern JJ, Berge S, Appel T, Reich RH, Kruger E (2000). Staged operations for basal cell carcinoma of the face. Br J Oral Maxillofac Surg.

[B28] Hallock GG, Lutz DA (2001). A prospective study of the accuracy of the surgeon's diagnosis and significance of positive margins in nonmelanoma skin cancers. Plast Reconstr Surg.

[B29] Hsuan JD, Harrad RA, Potts MJ, Collins C (2004). Small margin excision of periocular basal cell carcinoma: 5 year results. Br J Ophthalmol.

[B30] Fleischer AB, Feldman SR, Barlow JO, Zheng B, Hahn HB, Chuang TY (2001). The specialty of the treating physician affects the likelihood of tumor-free resection margins for basal cell carcinoma: results from a multi-institutional retrospective study. J Am Acad Dermatol.

[B31] Barry J, Foon S, Watson R (2004). A retrospective audit of the management of basal cell carcinoma in a dermatology department. Br J Dermatol.

[B32] Seidman JD, Berman JJ, Moore GW (1991). Basal cell carcinoma: importance of histologic discontinuities in the evaluation of resection margins. Mod Pathol.

[B33] Griffiths RW, Suvarna SK, Stone J (2005). Do basal cell carcinomas recur after complete conventional surgical excision?. Br J Plast Surg.

[B34] Smeets NWJ, Krekels GAM, Ostertag JU (2004). Surgical excision vs Mohs' micrographic surgery for basal cell carcinoma of the face: randomised controlled trial. Lancet.

[B35] Wilson AW, Howsam G, Santhanam V (2004). Surgical management of incompletely excised basal cell carcinomas of the head and neck. Br J Oral Maxillofac Surg.

[B36] Asadi AK, Alam M, Goldberg LH, Peterson SR, Silapunt S, Jih MH Efficacy of narrow-margin excision of well-demarcated primary facial basal cell carcinomas. J Am Acad Dermatol.

[B37] Goldberg DP (1997). Assessment and surgical treatment of basal cell skin cancer. Clin Plast Surg.

[B38] Griffiths a RW, Suvarna b SK, Stone b J (2007). Basal cell carcinoma histological clearance margins: an analysis of 1539 conventionally excised tumours. Wider still and deeper?. Journal of Plastic, Reconstructive Aesthetic Surgery.

[B39] Brodland DG, Zitelli JA (1992). Surgical margins for excision of primary cutaneous squamous cell carcinoma. J Am Acad Dermatol.

[B40] Rowe DE, Carroll RJ, Day CL (1992). Prognostic factors for local recurrence, metastasis, and survival rates in squamous cell carcinoma of the skin, ear, and lip. Implications for treatment modality selection. J Am Acad Dermatol.

[B41] Wolf DJ, Zitelli JA (1987). Surgical margins for basal cell carcinoma. Arch Dermatol.

[B42] Huang CC, Boyce SM (2004). Surgical margins for excision for basal cell carcinoma and squamous cell carcinoma. Semin Cutan Med Surg.

[B43] Epstein E (1973). How accurate is the visual assessment of basal cell carcinoma margins?. Br J Dermatol.

[B44] Seidman JD, Berman JJ, Moore GW (1991). Basal cell carcinoma: importance of histologic discontinuities in the evaluation of resection margins. Mod Pathol.

[B45] Griffiths RW (1999). Audit of histologically incompletely excised basal cell carcinomas: recommendations for management by re-excision. Br J Plast Surg.

[B46] Dzubow LM (1988). False negative tumor free margins following Mohs surgery. J Dermatol Surg Oncol.

[B47] Lang PG, Duncan IM, Hochman M (2004). Occurrence of subclinical tumor in excised facial subunits. Arch Facial Plast Surg.

[B48] Weinstock MA (1994). Epidemiologic investigation of nonmelanoma skin cancer mortality: The Rhode Island Follow- Back Study. J Invest Dermatol.

[B49] Rowe DE, Carroll RJ, Day CL (1992). Prognostic factors for local recurrence, metastasis, and survival rates in squamous cell carcinoma of the skin, ear, and lip. Implications for treatment modality selection. J Am Acad Dermatol.

[B50] Lund HZ (1965). How often does squamous cell carcinoma of the skin metastasize?. Arch Dermatol.

[B51] Khurana VG, Mentis DH, O'Brien CJ, Hurst TL, Stevens GN, Packham NA (1995). Parotid and neck metastases from cutaneous squamous cell carcinoma of the head and neck. Am J Surg.

[B52] Tavin E, Persky M (1996). Metastatic cutaneous squamous cell carcinoma of the head and neck region. Laryngoscope.

[B53] Bowman PH, Ratz JL, Knoepp TG, Barnes CJ, Finley EM (2003). Basosquamous carcinoma. Dermatol Surg.

[B54] Bogdanov-Berezovsky A, Cohen AD, Glesinger R, Cagnano E, krieger Y, Rosember L (2004). risk factor for incomplete excision for basal cell carcinomas. Acta Derm Venereol.

[B55] Leibovitch I, Huilgol SC, Selva D, Richards S, Paver R (2005). Basosquamous carcinoma. Treatment with Mohs Microgaphic Surgery. Cancer.

